# When Ablation Is Not the Answer: Acute Left Main Thrombosis Causing Incessant Ventricular Tachycardia Following Left Ventricular Assist Device Implant

**DOI:** 10.7759/cureus.1790

**Published:** 2017-10-21

**Authors:** Mohammad K Mojadidi, Ahmed N Mahmoud, Akram Y Elgendy, R. David Anderson

**Affiliations:** 1 Cardiology, Shands; 2 Medicine, University of Florida Health

**Keywords:** left ventricular assist device, acute coronary syndrome, thrombectomy, left main

## Abstract

Ventricular arrhythmia from aortic cusp thrombosis and coronary embolization is a rare complication of left ventricular assist device (LVAD) implantation. In this report, we present a case of acute left main and left anterior descending artery occlusion from embolic aortic cusp thrombi after LVAD implant. The patient presented with chest pain and incessant ventricular tachycardia post-LVAD implant. This was successfully treated by intracoronary thrombolysis, aspiration thrombectomy, and rheolytic thrombectomy. We present a rare case of successful percutaneous coronary intervention performed for incessant ventricular tachycardia in the setting of acute coronary thrombosis following LVAD implant.

## Introduction

Ventricular arrhythmia from aortic cusp thrombosis and acute coronary embolization is a rare complication of left ventricular assist device (LVAD) implantation. Successful percutaneous coronary intervention has rarely been reported [1]. Given the rarity of this entity, the optimal management of this complication is currently unknown. We present a case of incessant ventricular tachycardia from acute left main coronary artery thrombosis as a complication of LVAD implantation.

## Case presentation

**Figure 1:**
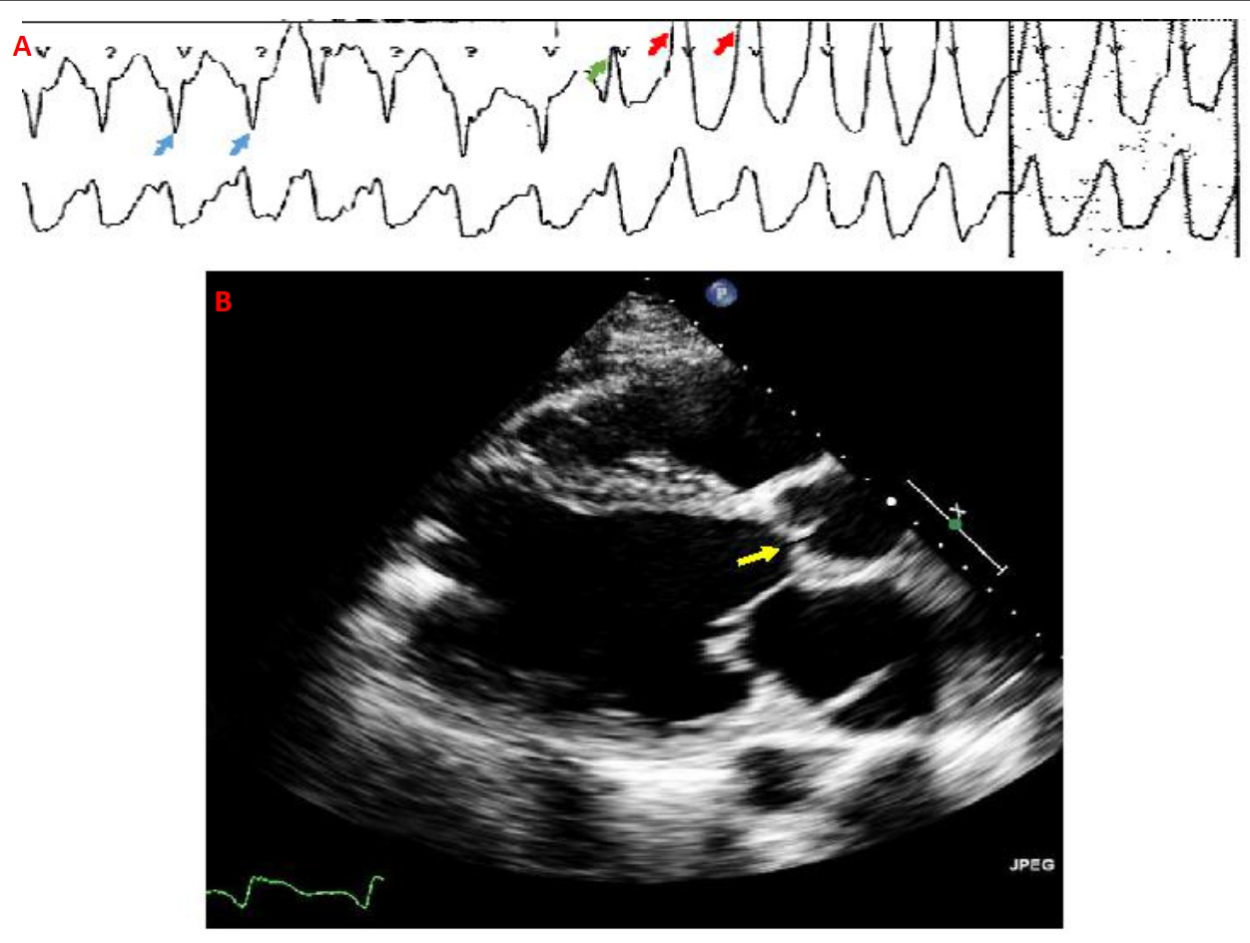
Telemetry (A) and Echocardiography (B) A) Telemetry strip showing ventricular tachycardia Blue arrows: paced rhythm, Green arrow: fusion beat, Red arrows: Ventricular tachycardia B) Left parasternal long-axis view showing lack of opening of both aortic cusps (yellow arrow) in systole

**Figure 2:**
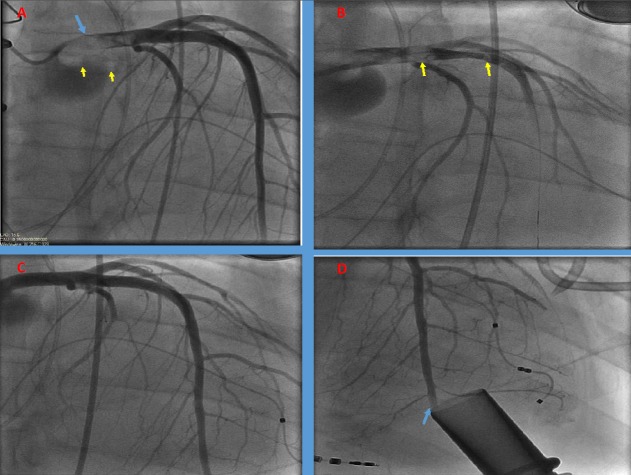
Coronary angiography pre- and post-intervention A) Pre-intervention angiography showing multiple thrombi in the left coronary cusp (yellow arrows) and the left main (blue arrow) B) Post wire insertion angiogram showing evidence of distal embolization of thrombi (yellow arrows) C) Proximal left main and left anterior descending artery angiography post-intervention D) Distal left anterior descending post-intervention (blue arrow)

A 41-year-old male with hypertension, ventricular tachycardia (VT) status post biventricular implantable cardioverter defibrillator (ICD), and non-ischemic cardiomyopathy with end-stage heart failure (left ventricular ejection fraction 10%) was admitted with decompensated heart failure. The patient was treated with diuresis and ionotropic support. Eleven days later, the patient received an LVAD Heartmate II (Thoratec, Pleasanton, California). On postoperative day 25, the patient experienced severe chest pain followed by multiple ICD shocks. Telemetry confirmed VT (Figure 1A). Medical therapy was initiated followed by intubation and sedation for incessant VT. Echocardiogram showed unobstructed LVAD cannula flow at the apex, severe right ventricular dilation, and hypokinesis; the aortic valve did not open during systole (Figure 1B). Coronary angiography illustrated a large thrombus burden in the left coronary sinus extending to the left main and proximal left anterior descending (LAD) artery (Figure 2A). A six French contralateral support (CLS) guiding catheter (Boston Scientific, Marlborough, Massachusetts, US) and a Prowater guidewire (Abbott Vascular, Santa Clara, CA, US) were advanced (Figure 2B). Aspiration thrombectomy was performed and multiple passes were made without success; thus, rheolytic thrombectomy was attempted, which was again unsuccessful. The guide catheter was advanced into the left main and aspiration was performed with a 20 cc syringe, yielding 1-2 x 6-8 mm pieces of organized thrombus, without improvement in the distal flow. Intra-coronary tPA injection, with repeated rheolytic thrombectomy, resulted in near resolution of the thrombus (Figure 2C) except at the distal LAD (Figure 2D). The patient was extubated the next day and remained free of chest pain and VT. He was later discharged with heart-failure clinic follow-up.

## Discussion

While the rate of serious, adverse events associated with LVADs has decreased with the development of newer devices and increased surgical experience, they have continued to occur and can be life-threatening [2]. Aortic root thrombosis can complicate the failure of aortic valve opening after an LVAD implant and has been described in the literature [3-5], even in the presence of anticoagulation [3]. Embolization of these thrombi can rarely cause acute left main thrombosis and incessant ventricular arrhythmias post-LVAD implant [6].

Given the rarity of this complication, optimal percutaneous management of this type of acute coronary thrombosis is unknown. In asymptomatic and hemodynamically stable patients, the current practice has been to intensify the anticoagulation and antiplatelet medical regimen, if bleeding risk is not excessive [6]. In this case presentation, we demonstrate that intracoronary tPA and rheolytic thrombectomy are feasible options for the percutaneous management of this life-threatening condition.

## Conclusions

Ventricular tachycardia from aortic cusp thrombosis and acute coronary embolization is a rare complication of LVAD implantation. Given its rarity, the optimal management of this complication is unclear. In this report, we presented a patient with incessant ventricular tachycardia from acute left main coronary artery thrombosis as a complication of LVAD implantation. The patient was successfully treated with percutaneous intracoronary tPA and rheolytic thrombectomy. Intracoronary tPA and rheolytic thrombectomy appear to be feasible options for managing unstable acute left main thrombosis following an LVAD implant.
